# Cis-Nerolidol Inhibits MAP Kinase and NF-κB Signaling Pathways and Prevents Epithelial Tight Junction Dysfunction in Colon Inflammation: In Vivo and In Vitro Studies

**DOI:** 10.3390/molecules28072982

**Published:** 2023-03-27

**Authors:** Vishnu Raj, Balaji Venkataraman, Shreesh K. Ojha, Saeeda Almarzooqi, Veedamali S. Subramanian, Basel K. Al-Ramadi, Thomas E. Adrian, Sandeep B. Subramanya

**Affiliations:** 1 Department of Physiology, College of Medicine and Health Sciences, United Arab Emirates University, Al-Ain P.O. Box 15551, United Arab Emirates; 2 Zayed Bin Sultan Center for Health Sciences, College of Medicine and Health Sciences, United Arab Emirates University, Al-Ain P.O. Box 15551, United Arab Emirates; 3 Department of Pharmacology and Therapeutics, College of Medicine and Health Sciences, United Arab Emirates University, Al-Ain P.O. Box 15551, United Arab Emirates; 4 Department of Pathology, College of Medicine and Health Sciences, United Arab Emirates University, Al-Ain P.O. Box 15551, United Arab Emirates; 5 Department of Medicine, University of California, Irvine, CA 92697, USA; 6 Department of Medical Microbiology and Immunology, College of Medicine and Health Sciences, United Arab Emirates University, Al-Ain P.O. BOX 15551, United Arab Emirates; 7 Department of Basic Medical Sciences, College of Medicine, Mohammed Bin Rashid University of Medicine and Health Sciences, Dubai P.O. Box 505055, United Arab Emirates

**Keywords:** nerolidol, RAW macrophages, MAPK, DSS colitis, Caco-2 cells, epithelial tight junction

## Abstract

Inflammation of the GI tract leads to compromised epithelial barrier integrity, which increases intestine permeability. A compromised intestinal barrier is a critical event that leads to microbe entry and promotes inflammatory responses. Inflammatory bowel diseases that comprise Crohn’s disease (CD) and ulcerative colitis (UC) show an increase in intestinal permeability. Nerolidol (NED), a naturally occurring sesquiterpene alcohol, has potent anti-inflammatory properties in preclinical models of colon inflammation. In this study, we investigated the effect of NED on MAPKs, NF-κB signaling pathways, and intestine epithelial tight junction physiology using in vivo and in vitro models. The effect of NED on proinflammatory cytokine release and MAPK and NF-κB signaling pathways were evaluated using lipopolysaccharides (LPS)-stimulated RAW 264.7 macrophages. Subsequently, the role of NED on MAPKs, NF-κB signaling, and the intestine tight junction integrity were assessed using DSS-induced colitis and LPS-stimulated Caco-2 cell culture models. Our result indicates that NED pre-treatment significantly inhibited proinflammatory cytokine release, expression of proteins involved in MAP kinase, and NF-κB signaling pathways in LPS-stimulated RAW macrophages and DSS-induced colitis. Furthermore, NED treatment significantly decreased FITC-dextran permeability in DSS-induced colitis. NED treatment enhanced tight junction protein expression (claudin-1, 3, 7, and occludin). Time-dependent increases in transepithelial electrical resistance (TEER) measurements reflect the formation of healthy tight junctions in the Caco-2 monolayer. LPS-stimulated Caco-2 showed a significant decrease in TEER. However, NED pre-treatment significantly prevented the fall in TEER measurements, indicating its protective role. In conclusion, NED significantly decreased MAPK and NF-κB signaling pathways and decreased tight junction permeability by enhancing epithelial tight junction protein expression.

## 1. Introduction

Inflammatory bowel diseases, comprised of Crohn’s disease (CD) and ulcerative colitis (UC), are chronic and progressive immune-mediated inflammatory conditions of the gastrointestinal (GI) tract that negatively affect epithelial barrier integrity [1,2]. Studies have demonstrated that a compromised intestinal epithelial barrier is associated with several intestinal disorders, including IBD [3,4]. The intestinal epithelium comprises a single layer of columnar epithelial cells (acting as a barrier between luminal contents), surface mucus layers, and the underlying immune-stromal-rich layer of lamina propria [4,5]. This epithelial layer acts as a physical barrier, providing a dynamic and selective permeability that prevents the entry of microbial pathogens, toxins, and antigens from the intestinal lumen into the lamina propria [6]. A breach in the intestinal barrier allows luminal microorganisms to enter the body, which in turn stimulates the immune system in the lamina propria [7]. The intestinal epithelial tight junction comprises multiple protein complexes localized at the apical end of the epithelial cell’s lateral side that regulate the paracellular pathway for the movement of substances. There are four types of integral transmembrane proteins: (i) occludin; (ii) claudins; (iii) junctional adhesion molecule (JAM); and (iv) tricellulin. Zonula occludins (ZO) are intracellular proteins primarily responsible for anchoring the transmembrane proteins into the peri-junctional actin cytoskeleton. Tight junctions play an essential role in intestinal homeostasis; therefore, their disruption or rearrangement is involved in the pathogenesis of inflammatory bowel diseases (IBD) [8]. Colonic biopsies from patients with colitis have been found to express reduced levels of epithelial tight junction proteins such as occludin, claudin-1, -3, -4, and -7, tricellulin, and JAMs [9]. Similarly, the downregulation of tight junction protein expression, including claudins, occludin, and ZOs, is also seen in experimental models of colon inflammation induced by dextran sodium sulfate [10,11].

A significant level of proinflammatory cytokines secreted by the intestinal epithelium during inflammation activates various intracellular kinases, including MAP kinases, during the development of IBD, and mediates the transcriptional regulation of multiple genes involved in IBD progression [7,12]. The transcription factor NF-κB is one of the downstream targets of MAP kinases. Activation of MAP kinases also causes an increase in intestinal paracellular permeability via the activation of myosin light chain kinase (MLCK) [13]. Therefore, it is conceivable that suppressing the MAP kinase pathway is beneficial in limiting the inflammatory response and protecting against barrier dysfunction.

Cis-Nerolidol (NRD) [3,7,11-trimethyl-1,6,10-dodecatrien-3-ol] is an aliphatic sesquiterpene commonly found in essential oils from plants with a floral odor. A high amount of nerolidol is found in oolong tea and Madeira wines and is also responsible for flavors of kiwifruit and strawberry fruits [14,15,16,17]. Based on this, the US Food and Drug Administration (FDA) has categorized nerolidol as “generally regarded as safe” (GRAS) and has been approved as a flavor enhancer that can be used in the food industry [18]. Recent studies have shown it has potent antioxidant, anti-inflammatory, anti-microbial, anti-cancer, and anti-ulcer activities [18,19]. We recently reported that NED significantly exhibited antioxidant activity by stimulating the Keap-1/Nrf2 signaling pathway and also showed potent antioxidant and anti-inflammatory activity in colon inflammation using in vivo and in vitro models [2]. However, the molecular mechanisms mediating the anti-inflammatory activity and its role in modulating intestinal tight junction physiology have not been investigated. In the present study, we investigated the role of NED on the MAPK and NF-κB signaling pathways and intestine epithelial barrier function using in vivo and in vitro models.

## 2. Results

### 2.1. Effect of NED on Colon Length and Disease Activity Index (DAI)

**Figure 1:**
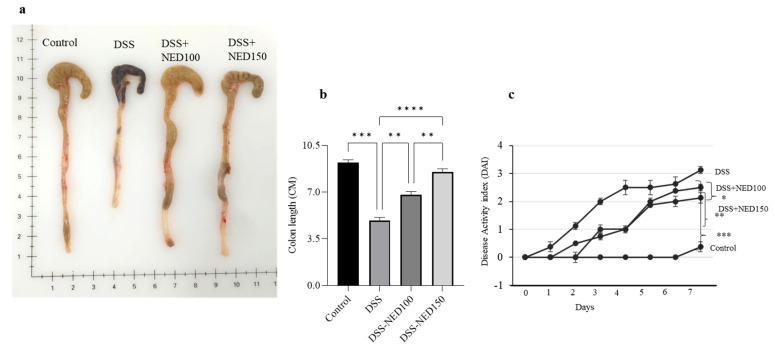
Effect of NED on colon length and DAI. DSS Administration significantly shortened colon length and increased DAI compared to controls. NED significantly prevented the shortening of the colon and DAI (**a**–**c**). Data was obtained from n = 8 animals. The results are expressed as mean ± SEM. * *p* ≤ 0.05, ** *p* ≤ 0.01, *** *p* ≤ 0.001, and **** *p* ≤ 0.0001.

The effect of NED was evaluated in DSS-induced colitis mice on colon length and DAI. It is well established that DSS administration increases DAI and shortens the colon. A significant increase in DAI and shortening of the colon was observed in the DSS group compared to the controls (Figure 1). NED treatment significantly decreased DAI and the shortening of the colon (a–c). These findings are similar to our previous study; however, these new experiments were intended to establish the role of NED on MAP kinase and tight junction protein function.

### 2.2. Effect of NED on Cell Viability and Proinflammatory Cytokine Profiles and COX-2 and iNOS Protein Expression in LPS-Stimulated RAW 264.7 Macrophages

**Figure 2:**
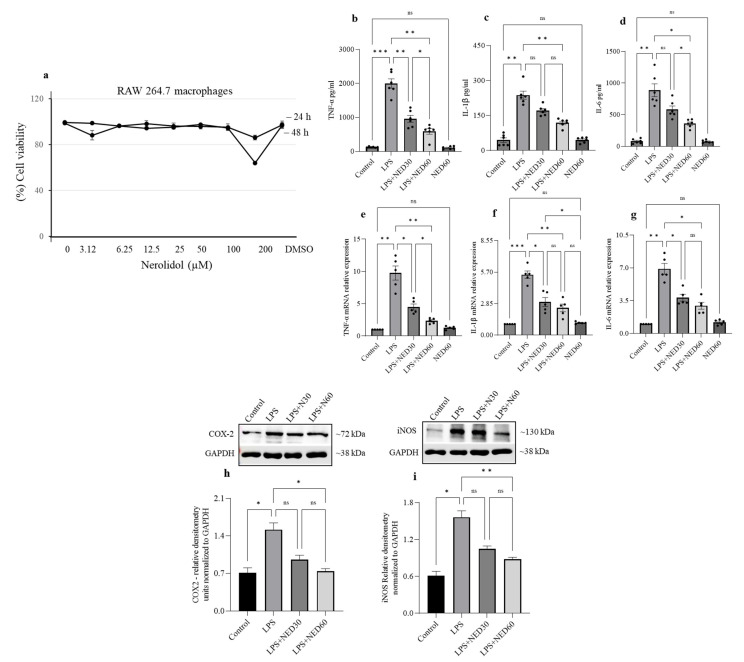
Effect of NED on cell viability and proinflammatory cytokines and COX-2 and iNOS protein expression in LPS-stimulated RAW 264.7 macrophages. (**a**) Cell viability was determined through treatment with various concentrations of NED. LPS-stimulated macrophages showed a significant increase in the release of proinflammatory cytokine protein (**b**–**d**) and mRNA expression (**e**–**g**) of TNF-α, IL-1β, and IL-6. NED pre-treatment significantly prevented this. (**h**,**i**) LPS stimulation significantly increased COX-2 (**h**) and iNOS (**i**) protein expression. NED treatment prevented the further rise of COX-2 and iNOS protein expression induced by LPS. Data were obtained from n = 5 separate cell culture samples for ELISA and mRNA expression. Densitometry values are expressed as means ± SEM (n = 3). The results are expressed as mean ± SEM. * *p* ≤ 0.05, ** *p* ≤ 0.01, and *** *p* ≤ 0.001 and ns indicates not significant.

The concentration-dependent cytotoxic effects of NED were investigated using RAW 264.7 macrophage cells treated with different concentrations of NED in order to establish a non-toxic dose range for treatment. Cells were treated with varying concentrations of NED ranging from 0 µM to 200 µM for 24 and 48 h (h). NED treatment did not show significant cell death at 24 and 48 h (Figure 2a). Based on these observations, doses of 30 and 60 µM NED were selected for subsequent investigations in RAW macrophages. To examine the effect of NED on LPS-stimulated proinflammatory cytokine release (TNF-α, IL-1β, and IL-6) and mRNA expression, the RAW 264.7 macrophage cells were pre-incubated with NED (30 and 60 µM) for 24 h, followed by LPS-stimulation (1 μg/mL) for 6 h. LPS stimulation significantly increased proinflammatory cytokine production in RAW macrophages in untreated controls. NED pre-treatment at 30 and 60 µM significantly decreased the LPS-induced increase in proinflammatory cytokines, TNF-α, IL-1β and IL-6 release (b–d), and expression of their mRNAs (e–g). However, NED (60 µM) alone showed no significant effect compared to the untreated control. Therefore, the NED treatment alone group was omitted from the subsequent experiments. LPS significantly increased expression of COX2 and iNOS proinflammatory mediators at the protein levels. NED pre-treatment significantly reduced COX2 and iNOS levels in LPS-stimulated RAW macrophages (h, i).

### 2.3. Effect of NED Pre-Treatment on the Phosphorylation of MAPK and NF-κB Proteins in LPS-Stimulated RAW Macrophages

**Figure 3:**
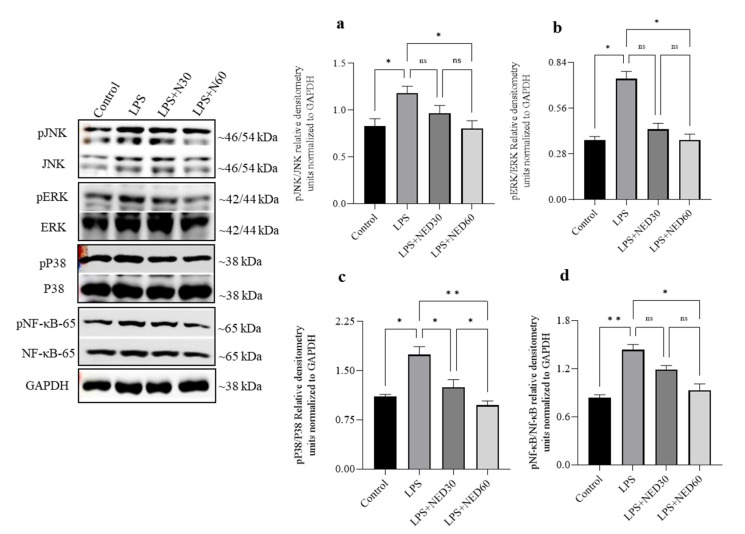
Effect of NED pre-treatment on the phosphorylation of MAPK and NF-κB proteins in LPS-stimulated RAW Macrophages. NED treatment significantly prevented the phosphorylation and activation of p38, JNK, and ERK1/2 MAP kinases and NF-κB induced by LPS in RAW macrophages (**a**–**d**). Densitometry values are expressed as means ± SEM (n = 4). ** *p* ≤ 0.01, * *p* ≤ 0.05, and ns indicates not significant.

LPS stimulation significantly increased the phosphorylation of JNK, ERK^1/2^, and p38 MAP kinases in RAW macrophages. NED pre-treatment significantly reduced the phosphorylation of all three MAP kinases (a–c). Similarly, stimulation of RAW macrophages with LPS resulted in a significantly enhanced phosphorylation of NF-κB (d). NED pre-treatment (60 µM) significantly inhibited the phosphorylation of NF-κB (d). No significant effect on the phosphorylation of NF-κB was observed with 30 µM NED dose. These results indicate that NED significantly inhibited the phosphorylation of MAP kinase and the NF-κB signaling pathways to prevent their activation (Figure 3a–d).

### 2.4. Effect of NED on the Phosphorylation of MAP Kinase and NF-κB Signaling Pathways in DSS-Induced Colitis Mice

**Figure 4:**
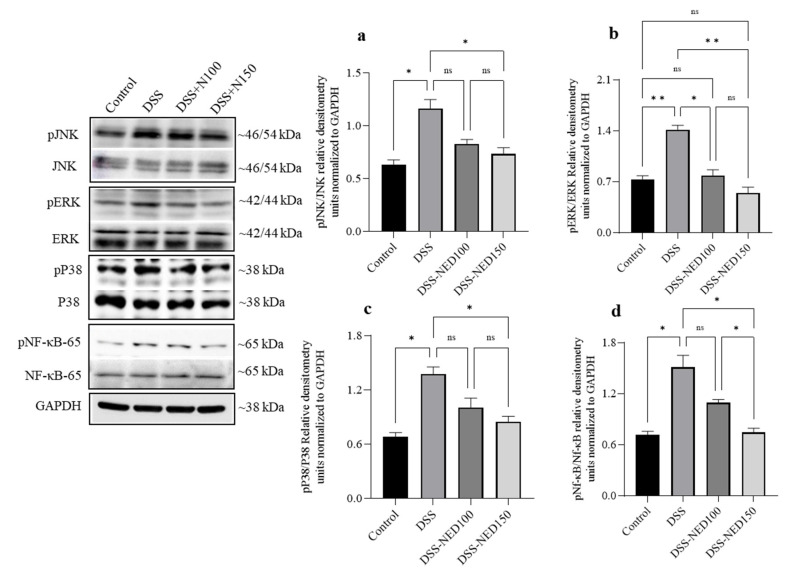
Effect of NED on the phosphorylation of MAP kinase and NF-κB signaling pathways in DSS-induced colitis mice. Western blot experiments showed that NED treatment significantly inhibited the DSS-induced phosphorylation of JNK, ERK^1/2^, and p38 MAP kinases and NF-κB in mice (**a**–**d**). Densitometry values are expressed as means ± SEM (n = 4). * *p* ≤ 0.05, ** *p* ≤ 0.01, and ns indicates not significant.

The DSS-induced colitis model was used to evaluate the role of NED on MAPK and NF-κB signaling pathways in an in vivo model of colon inflammation. The DSS administration in mice enhanced the phosphorylation of JNK, ERK^1/2^, and p38 MAP kinases. NED treatment significantly suppressed the phosphorylation of these MAP kinases (Figure 4). Nuclear translocation of NF-κB is a critical event that regulates the production of proinflammatory cytokines. Phosphorylation/activation of NF-κB leads to the nuclear translocation of NF-κB. DSS administration significantly increased the phosphorylation of NF-κB. NED treatment (60 μM) significantly prevented the NF-κB phosphorylation (d). These results indicate that NED significantly decreases the phosphorylation of p38, JNK, and ERK^1/2^ MAP kinases and NF-κB signaling pathways in the in vivo model of colon inflammation.

### 2.5. Effect of NED on Colon Histology, Colon Permeability, and Colon Epithelial Tight Junction Protein Expression in DSS-Induced Colitis Mice

**Figure 5:**
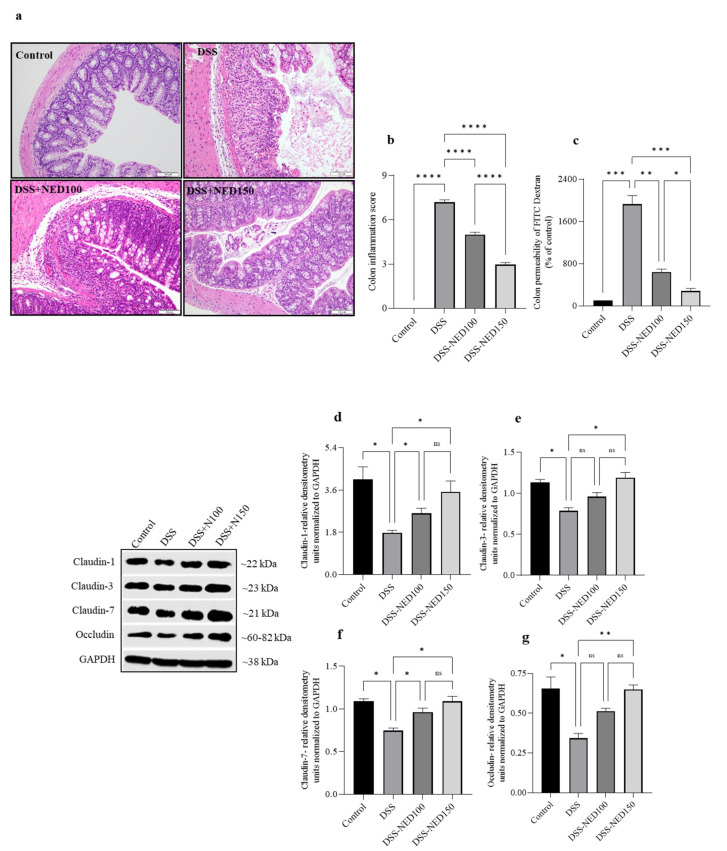
Effect of NED on colon histology, colon permeability, and colon epithelial tight junction protein expression in DSS-induced colitis mice. DSS administration significantly altered colon microarchitecture with an increase in colon inflammation score, and NED prevented it (**a**,**b**). DSS administration significantly enhanced FITC-dextran permeability (**c**). NED treatment at 100 and 150 mg/kg doses significantly inhibited the DSS-induced increased permeability of FITC-dextran. (**d**–**g**) DSS significantly downregulated claudin-1, -3, -7, and occludin protein expression. NED treatment significantly increased tight junction protein expression. Data expressed as mean ± SEM (n = 6). * *p* ≤ 0.05, ** *p* ≤ 0.01, *** *p* ≤ 0.001, **** *p* ≤ 0.0001, and ns indicates not significant.

The administration of DSS led to the destruction of the normal colonic epithelial morphology and crypt distortion, as well as an increase in the number of infiltrating cells in the submucosal compartment. NED treatment significantly protected colon epithelial morphology and decreased colon inflammation scores (Figure 5). Intestinal epithelial tight junction proteins play a critical role in maintaining intestinal barrier integrity and paracellular permeability. FITC-Dextran permeability serves as a surrogate marker to assess colon permeability. We used 4-kDa dextran (non-digestible) tagged with fluorescein isothiocyanate (FD-4). DSS administration significantly increased FITC-dextran permeability, indicating intestinal barrier dysfunction. NED treatment significantly prevented FITC-dextran permeability, indicating its protective role in barrier dysfunction induced by DSS colitis (Figure 5). To further understand the mechanism behind NED-mediated decrease in FITC permeability, a Western blot analysis of tight junction proteins was carried out. Compared to the control group, the tight junction proteins claudin-1, -3, -7, and occludin were significantly downregulated in the colon homogenate of mice administered with DSS. However, the higher dose of NED (150 mg/kg) treatment prevented downregulation and even enhanced tight junction protein expression in the DSS-induced colitis group (Figure 5).

### 2.6. Effect of NED on Cell Viability and Proinflammatory Chemokine mRNA Expression in LPS-Stimulated Caco-2 Cells

**Figure 6:**
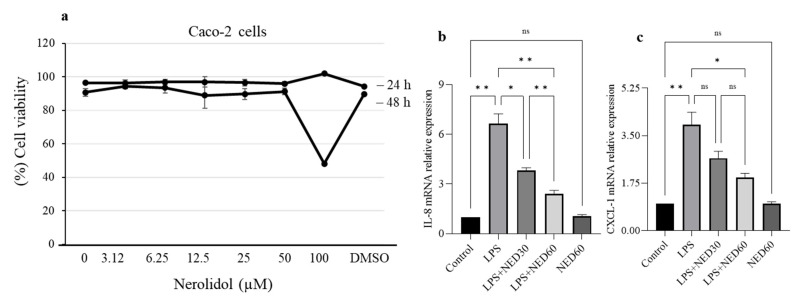
Effect of NED on cell viability and proinflammatory chemokine mRNA expression in LPS-stimulated Caco-2 cells. The concentration-dependent cytotoxic effects of NED were evaluated for 24 h and 48 h (**a**). LPS stimulation significantly increases proinflammatory chemokine (IL-8 & CXCL-1) mRNA expression (**b**,**c**). NED treatment (30 and 60 µM) significantly decreased it. Data expressed as mean ± SEM (n = 6). * *p* ≤ 0.05, ** *p* ≤ 0.01, and ns indicates not significant.

The effect of NED on mRNA expression of proinflammatory chemokines was evaluated using the Caco-2 cell line. Caco-2 cells were treated with different concentrations of NED from 0 µM to 100 µM for 24 h and 48 h. NED did not induce a cytotoxic effect on cell viability until 100 µM dose at 24 h (Figure 6). Based on these observations, 30 and 60 µM concentrations were selected for subsequent experiments. Stimulating Caco-2 cells with LPS (100 μg/mL) resulted in a significant increase in the mRNA expression of proinflammatory chemokines, IL-8, and CXCL-1. NED pre-treatment significantly downregulated IL-8 and CXCL-1 mRNA in LPS-stimulated Caco-2 cells (Figure 6). However, NED alone at 60 µM concentration did not show any significant effect compared to the control.

### 2.7. Effect of NED on Caco-2 Transepithelial Electrical Resistance (TEER) Measurements

**Figure 7:**
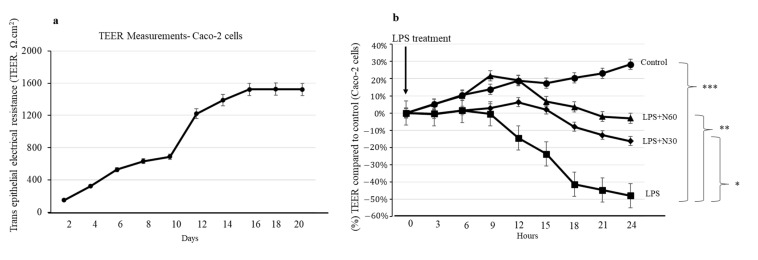
Effect of NED on Caco-2 transepithelial electrical resistance (TEER) measurements. (**a**) TEER measurements showed a steady increase in the values up to 16 days; (**b**) LPS stimulation significantly reduced the TEER measurements. Preincubation with NED significantly prevented further drop in TEER values. Data are expressed as mean ± SEM (n = 6). *** *p* ≤ 0.001, ** *p* ≤ 0.01, * *p* ≤ 0.05, and ns = not significant.

The epithelial barrier integrity of Caco-2 monolayers was evaluated by measuring TEER values daily. An increase in TEER values is an indicator of healthy TJ formation. TEER showed a steady increase daily until it reached a plateau over 14–15 days (Figure 7). Caco-2 monolayers showing ~1500 Ω.cm^2^ were used for TEER measurement studies. These monolayers were pre-treated with NED for 24 h and subsequently challenged with LPS (100 μg/mL). After the LPS challenge, TEER measurements were recorded every 3 h using an EVOM^2^ m for 24 h. A drop in TEER values was recorded after 12 h of the LPS challenge, continued to drop for 24 h, and showed a 50% decrease compared to the control (Figure 7). These results indicate that epithelial barrier integrity is compromised upon the LPS challenge. NED pre-treatment in LPS-stimulated Caco-2 monolayer significantly prevented the decrease in TEER at both the doses (30 μm and 60 μM) (Figure 7). These results indicating that NED pre-treatment prevented LPS-stimulated decrease in TEER is an indicator of its protective role.

## 3. Discussion

Our previous study demonstrated that NED mitigated the proinflammatory response and activated the Keap-1/Nrf2 signaling pathway to induce potent antioxidant effects using in vivo and in vitro models of colon inflammation [2]. In the present study, we investigated the role of NED in mitigating potent proinflammatory signaling pathways such as MAP kinases and NF-κB. We used RAW 264.7 murine macrophage cells stimulated by lipopolysaccharide (LPS) as an in vitro model and DSS-induced colitis as an in vivo model to understand how NED affects MAP kinase and NF-B signaling pathways. We also used in vivo and in vitro models to investigate the effect of NED on the colon epithelial tight physiology, which is dysregulated in inflammation.

MAPKs comprised of ERKs, JNKs, and p38 MAPKs are activated by various stimuli and play a significant role in inflammatory responses [20]. Both preclinical and clinical studies show that the activation of MAPKs promotes the release of proinflammatory cytokines [8,21]. Evidence suggests that inhibition of MAP kinases with specific inhibitors can be beneficial in the clinical setting of IBD [7,8,22]. Experimental evidence also indicates that inhibition of p38 MAPKs and JNK resulted in decreased proinflammatory cytokine release and also decreased NF-κB phosphorylation in DSS colitis [23,24]. The JNK inhibitor SP600125 was also found to protect murine colitis by downregulating the release of proinflammatory cytokines [12]. MAPK inhibition in an in vitro colon inflammation model showed a similar decrease in the proinflammatory cytokines IL-6 and IL-8 levels [25]. However, few studies have investigated the role of ERKs in IBD. ERK^1/2^ phosphorylation increased in IBD and DSS-induced colitis, and this enhanced proinflammatory cytokine release [7,26]. Increased IL-6 production in UC is mediated by ERK^1/2^. The inhibition of ERK^1/2^ phosphorylation significantly reduced IL-6 release [27]. Previous studies have shown that NED downregulated MAPK signaling pathways in doxorubicin-induced cardiotoxicity and LPS-induced lung injury [28,29]. Our results indicate that NED both decreased phosphorylation of the MAPK signaling proteins in LPS-stimulated RAW macrophages and in DSS-induced colitis contributed to decreased proinflammatory cytokine release. NF-κB mediates the transcription of several proinflammatory genes and plays a crucial role in regulating inflammatory processes [30]. Previous studies have also shown that NED ameliorated cardiac and neuroinflammation by downregulating NF-κB signaling pathways [31,32]. Our results also indicate that NED significantly reduced the phosphorylation of the transcription factor NF-κB in both in vivo and in vitro models.

The intestinal mucosal epithelium has a regularly aligned layer of epithelial cells with an underlying lamina propria and muscularis mucosa. The intestinal epithelium cells absorb water, electrolytes, and nutrients and maintain a physical barrier [33]. The inflammatory responses observed in UC and CD disrupt intestinal epithelial barrier integrity that contributes to the development of a leaky gut. Inflammation is the critical event that affects intestine barrier integrity and allows the entry of luminal antigens into the lamina propria via the paracellular pathway due to its compromised barrier [34]. The apical junctional complex comprises tight junction proteins and helps to regulate the paracellular permeability between two adjacent epithelial cells. The adherence junction located below/along with tight junction regulates cell–cell signaling, and desmosomes stabilize the epithelia [35].

The toxic effect of the sulfate moiety of DSS disrupts the barrier integrity and allows the entry of luminal antigens into the mucosa. This is followed by the activation of the innate immune response via the recruitment of inflammatory cells (neutrophils and macrophages). DSS modulates the expression of claudins-1, -3, -7, -8, and occludin in the distal colon and increases intestinal permeability. In the mouse model, DSS decreases claudin-1 expression [36,37,38].

Intestinal epithelial barrier integrity, or intestinal permeability, can be evaluated by administering macromolecules that cannot be digested and can permeate through a compromised colon epithelial barrier. This is considered to be the most reliable model for in vivo analysis of intestinal permeability analysis [39]. Several compounds can be used to determine intestinal permeability, including radioisotopes, polyethylene glycols, and sugars [40]. In the present study, FITC-dextran (4 kDa molecular weight) was used as a marker to assess barrier integrity. Due to its macro size, FITC-dextran does not cross the intestinal barrier under physiological conditions. However, it can diffuse through the barrier and be detected in the blood if barrier integrity is compromised. FITC-dextran takes about 3 h to reach the colon after passing through the GI tract [39]. The plasma concentration of FITC-dextran indicates the extent of intestinal barrier permeability. Results showed that DSS administration significantly increased FITC-dextran levels in serum compared to control animals. However, the NED treatment significantly prevented the increase in serum FITC-dextran concentration, indicating its role in protecting barrier dysfunction. This effect of NED can be attributed to enhanced tight junction protein expression. Previous studies have demonstrated that dietary phytochemicals such as quercetin, naringenin, and resveratrol increase tight junction proteins and reduce barrier dysfunction in experimental colitis. [41,42,43]. Furthermore, the role of NED on the physiology of barrier integrity was also assessed by measuring TEER in an in vitro model using Caco-2 (human colorectal adenocarcinoma) cells [44] and can be employed for colon inflammation studies. NED also significantly decreased the LPS-induced chemokines mRNA expression in Caco-2 cells. A significant drop in TEER was found over 24 h post-LPS-stimulation. This decrease in TEER was significantly reduced by NED pre-incubation, suggesting a protective role in maintaining barrier integrity.

Multiple previous studies have linked the causal effects of the MAPK and NF-κB pathways to colonic inflammation in the DSS mouse model [45,46,47,48]. MAP kinase signaling also plays a significant role in regulating paracellular permeability [49]. Myosin light chain (MLC) phosphorylation by myosin light chain kinase (MLCK) regulates cellular actomyosin contractions, a key step in maintaining barrier integrity by opening paracellular pathways [50]. TNF-α increases MLCK phosphorylation and, thus, enhances paracellular permeability. A previous study showed that inhibiting MLCK improved barrier function in TNF-α stimulated intestinal epithelial cells [51]. Previous studies reported that TNF-α suppresses occludin promoter activity and causes ZO-1 and claudin-1 rearrangement [52,53]. Similarly, SP600125, a JNK inhibitor, was shown to reduce DSS-induced JNK phosphorylation and prevent intestinal barrier disruption by increasing ZO-1 and occludin protein expression [54]. A variety of inflammation inhibitors in the DSS colitis model have been shown to attenuate inflammation as well as inhibit the activation of MAPK and NF-κB [45,46,47,48]. In these studies, the direct link between the activation of MAPK and NF-κB with mucosal inflammation has been shown using pharmacological agents, such as the MEK inhibitor, UO126 [45]; an inhibitor of mitogen-activated protein kinase phosphatase-1, NSC 95397 [46]; the dual MAPK/NF-κB inhibitor, Asperuloside; the JNK inhibitor, SP600125 [47]; and the MAPK agonist, BIM-23A760 [48]. Furthermore, the anti-TNF-α antibody adalimumab prevented increased activation of NF-κB, accompanying the decline in transepithelial electrical resistance (TEER) in cellular model systems. Pharmacological inhibition of NF-κB signaling partially prevented the TNF-α-induced TEER [55]. It was also reported that curcumin could prevent gut-derived bacterial LPS translocation into the circulation by protecting intestinal epithelial barrier integrity. Its effect was attributed to its inhibition of IL-1β signaling. IL-1β was reported to increase p38 MAPK phosphorylation, enhancing MLCK expression and paracellular permeability [56]. With the strength of all of this evidence, it can be concluded that the suppressive effects of nerolidol on inflammation, and the decrease in tight junction permeability, by enhancement of epithelial tight junction protein expression, is mediated by suppression of the MAPK and NF-κB signaling pathways.

## 4. Materials and Methods

### 4.1. Chemical, Reagents, and Cells

Dextran sulfate sodium (DSS) (Molecular weight 36,000–50,000 Da), was purchased from Sigma-Aldrich (St. Louis, MO, USA). Cis-Nerolidol, hexadecyltrimethylammonium bromide (HTAB), and ortho-dianisidine dihydrochloride (ODD) were purchased from Sigma-Aldrich (St. Louis, MO, USA). IL-6, IL-1β, and TNF-α ELISA kits were purchased from R&D systems (Minneapolis, MN, USA). The reverse transcription kit was procured from Applied Biosystems (Foster City, CA, USA). Evagreen 5X mastermix from Solis Bio-Dyne (Tartu, Estonia) provided the mastermix, and Macrogen Inc. (Seoul, Republic of Korea) supplied primers for quantitative RT-PCR. The protease and phosphatase inhibitors were procured from Thermo-Scientific (Rockford, IL, USA). The antibodies (JNK, p-JNK, ERK, p-ERK, p38, p-p38, Nf-κB, p-Nf-κB, claudin-1, and occludin) were purchased from Santacruz Biotechnology (Dallas, TX, USA). Caludin-3 and -7 antibodies were purchased from Thermo-Scientific (Rockford, IL, USA). Other reagents were obtained from the suppliers listed in our previous publication [57]. The RAW 264.7 macrophages and Caco-2 cells were obtained from the American Type Culture Collection (Manassas, VA, USA).

### 4.2. Animals

C57BL/6J mice (12 weeks old) weighing 25–30 g were procured from the central animal facility, CMHS, at UAE University. Two animals per cage were kept for each experiment 1 week before the start of the experiment for acclimatization. The animals were kept at a temperature of 23 ± 1 °C, on a 12-h light-dark cycle, and at a humidity of 50–60%. Food and water were provided ad libitum. The UAEU Institutional Animal Ethical Committee approved the current study (approval # ERA_2017_5599).

### 4.3. Experimental Design

Mice were randomly allocated to four groups with eight animals in each group used for in vivo studies. Group I: untreated control—vehicle treated (sunflower oil). Group II: DSS alone. Group III: DSS + Nerolidol (NED) (100 mg/kg body weight/day). Group IV: DSS + NED (150 mg/kg body weight/day). DSS (3%) was prepared freshly every day in autoclaved drinking water. At the end of (starting of the day considered as ‘0′ day and treatment ended on 7th day). After the 8-day treatment protocol, the animals were euthanized using pentobarbital overdose (100 mg/kg body weight).

### 4.4. Disease Activity Index (DAI), Colon Length, and Colon Histology

Colons, including the caecum, were surgically removed and photographed with a scale, and colon length was measured. DAI was calculated as mentioned previously [2]. Colons were then cleaned with ice-cold saline to remove fecal content. As described previously, a portion of the colon was fixed in 10% formaldehyde and processed for hematoxylin and eosin (H&E) staining [2]. The remaining colon mucosal layer was scraped to separate it from the muscle layer, snap-frozen immediately using liquid nitrogen, and stored at −80 °C until further use. After H&E staining, the microarchitecture was assessed for colon epithelium integrity, crypt distortion, submucosal edema, and submucosal infiltrating cells to construct colon inflammation scores by a clinical pathologist (Dr. Saeeda Almarzouqui, co-author in this study). All the histology samples were blinded for the histopathological evaluations.

### 4.5. Intestinal Permeability

Intestinal permeability was assessed following oral administration of FITC-dextran 4 kDa (Sigma-Aldrich, St. Louis, MO, USA). Mice were orally gavaged with FITC-dextran (40 mg/100 g body weight) 4 h before sacrifice after overnight fasting. Whole blood was obtained from the abdominal aorta at the time of sacrifice, and FITC-dextran measurements were performed in triplicate using fluorescence. A standard curve was created using FITC-dextran dilutions in PBS, and the absorption of 100 μL of serum or the standard was measured in a plate reader at 488 nm.

### 4.6. RNA Extraction and Real-Time RT-PCR

The RNA extraction from colonic mucosa and conversion into cDNA and real-time PCR were performed as described previously [2]. The 18s gene was used as an internal reference gene in the present study. The change in CT values was calculated using delta delta CT method (2^−ΔΔCT^) [58]. Primers used for real-time PCR analysis and the conditions to obtain PCR amplicons were the same as described in our previous publications [2,57].

### 4.7. Western Blot

Colonic mucosa frozen samples were homogenized in RIPA buffer dissolved with a protease and phosphatase inhibitor cocktail using a bead homogenizer as described previously [2]. The undiluted homogenate protein concentrations were determined using Pierce BCA Protein Assay Kit (Cat#23225, Thermo-Scientific, Waltham, MA, USA). A total of 20 µg (colon tissue and RAW 264.7 macrophages) of proteins were resolved using sodium dodecyl sulfate-polyacrylamide gel electrophoresis (SDS-PAGE) using 8–12% gels, and subsequently transferred onto PVDF membrane. These PVDF membranes were immune blotted using specific antibodies. GAPDH proteins were used as an internal control to normalize with the protein of interest. The primary antibodies used for the Western blot analysis were at the following concentrations: JNK, p-JNK, ERK, p-ERK, p38, p-p38, Nf-κB, p-Nf-κB, claudin-1, -3, -7 and occludin (1:1000). The secondary antibody GAPDH was used at 1:5000 concentration. Western blot bands were densitometrically examined using the free software ImageJ. In order to perform optical density (OD) calculations, the images captured by Azure Sapphire™ biomolecular imager (Azure biosystems, Dublin, OH, USA) were transformed in to 8-bit format. Each band was chosen individually, circumscribed using the rectangle ROI selection, and the peak area was then measured. The graph displays the bands’ measured pixel intensities using ImageJ.

### 4.8. RAW Macrophages and Caco-2 Cell Culture

RAW 264.7 macrophages and Caco-2 cells derived from colorectal adenocarcinoma were cultured at 37 °C and 5% CO_2_ in a humidified incubator in high-glucose DMEM, containing 100 U/mL penicillin, 100 µg/mL streptomycin, and 10% (*v/v*) heat-inactivated fetal bovine serum (FBS). Cultured macrophages were seeded (1.5 × 10^5^ cells per well) onto 6-well plates 24 h before treatment. Nerolidol (30 and 60 μM) was pre-treated for 24 h for all the cell culture protocols, followed by LPS (1 μg/mL for macrophages and 100 μg/mL for Caco-2 cells) stimulation for 6 h. After treatment, the medium was collected from the macrophages to estimate secreted inflammatory cytokines (IL-6, IL-1β, and TNF-α). RAW macrophages were collected with RIPA buffer added with protease inhibitors for Western blot. Both RAW macrophages and Caco-2 cells were collected and suspended in Trizol reagent for RNA isolation and proinflammatory cytokines and proinflammatory chemokine (IL-8 and CXCL-1) mRNA studies. The mRNA expression studies were carried out as described previously [2].

### 4.9. Cell Viability Assay

RAW 264.7 macrophages and Caco-2 cells (5000 cells/well) were seeded onto 96 well plates and treated with a range of nerolidol concentrations (0, 3.12, 6.25, 12.5, 25, 50, and 100 µM) for 24 h and 48 h. According to the manufacturer’s instructions, cell viability was determined using the Cell Titer-Glo^®^ viability kit (Cat#G7571, Promega, Madison, WI, USA) after the indicated treatment period. Luminescence was measured using a Tecan (Infinite^®^ 200 PRO, Männedorf, Switzerland) multimode plate reader. Data are represented as percent of viable cells (quantified by ATP content) in the nerolidol-treated groups compared with the untreated control group.

### 4.10. Trans-Epithelial Electrical Resistance (TEER) Measurements

Caco-2 cells were grown on a 12-well Transwell system, and the changes in TEER were determined using an epithelial volt-ohm meter (EVOM^2^, World Precision Instruments, Sarasota, FL, USA). The TEER measurements were recorded every day for 15 days until the value reached 1500 Ω cm^2^. Electrical resistance was measured until similar values were recorded on three consecutive measurements. Values were corrected for background resistance due to the membrane insert and calculated as Ω cm^2^. The Caco-2 epithelial resistance of 1500 Ω cm^2^ and above was the starting point of NED pre-incubation with different doses (30 and 60 μM) for 24 h, followed by LPS stimulation for 6 h in the experimental group; in the control group, only NED was incubated without LPS stimulation.

### 4.11. Statistical Analysis

Statistical analysis was performed using GraphPad Prism (version 9) software (San Diego, CA, USA). Group data were compared by one-way analysis of variance (one way-ANOVA). For multiple comparisons, the nonparametric Tukey’s post hoc test was used. Data represented as mean ± SEM. *p*-value < 0.05 are considered statistically significant.

## 5. Conclusions

NED significantly inhibited LPS-induced RAW macrophage proinflammatory cytokines and mediators. NED inhibited MAP kinase and NF-κB signaling pathways inLPS-stimulated RAW macrophages and DSS-induced colitis. NED protects against colon barrier dysfunction by decreasing colon permeability and enhancing expression of key tight junction protein expression in DSS-induced colitis. NED also prevents the decrease in LPS-stimulated TEER measurements. Therefore, NED is a promising lead compound for therapeutic development in colitis.

## Data Availability

Data is available upon request from the corresponding author.

## Sample Availability

Samples are not available. It is the property of the university.
